# Cardiac MRI-derived mean right atrial pressure and its prognostic importance

**DOI:** 10.1136/openhrt-2025-003216

**Published:** 2025-06-22

**Authors:** Tom Alexander Howard Newman, Gareth Matthews, Hosamadin Assadi, Rui Li, Ciaran Grafton-Clarke, Zia Mehmood, Bahman Kasmai, Chris Sawh, Liang Zhong, Samer Alabed, Joao L Cavalcante, Ross J Thomson, Nay Aung, Rob J van der Geest, Andrew J Swift, Pankaj Garg, The ASPIRE Consortium

**Affiliations:** 1 Division of Clinical Medicine, The University of Sheffield School of Medicine and Population Health, Sheffield, UK; 2 Sheffield Teaching Hospitals NHS Foundation Trust, Sheffield, UK; 3 NIHR Biomedical Research Centre, Sheffield Teaching Hospitals NHS Foundation Trust, Sheffield, UK; 4 Norfolk and Norwich University Hospitals NHS Foundation Trust, Norwich, UK; 5 Department of Cardiovascular and Metabolic Health, University of East Anglia, Norwich, UK; 6 National Heart Centre Singapore, Singapore; 7 Medical School, Duke-National University of Singapore, Singapore; 8 Minneapolis Heart Institute, Minneapolis, Minnesota, USA; 9 Queen Mary University of London, London, UK; 10 Barts Health NHS Trust, London, UK; 11 Leiden University Medical Center (LUMC), Leiden, The Netherlands

**Keywords:** heart failure, magnetic resonance imaging, pulmonary arterial hypertension

## Abstract

**Background:**

Right atrial pressure (RAP) is a key variable that cardiac MRI (CMR) cannot currently measure. We aimed to develop a model to estimate mean RAP (mRAP) using CMR and assess the prognostic value of CMR-derived mRAP in an independent patient cohort.

**Methods:**

The derivation cohort consisted of patients investigated for heart failure symptoms with right heart catheterisation and CMR. Right atrial and ventricular CMR measurements were correlated with invasive mRAP to inform multivariable linear regression models incorporating patient characteristics. CMR-derived mRAP was tested as a predictor for clinical outcomes (lower-limb oedema, heart failure hospitalisation and all-cause mortality) on an independent cohort of patients receiving CMR. Both cohorts were derived from hospital registries.

**Results:**

In the derivation cohort (n=672), invasive mRAP was >8 mm Hg in 56% of patients. Right atrial end-systolic volume (RAESV) had the strongest correlation with invasive mRAP (Pearson’s coefficient 0.58, p<0.01). RAESV was as accurate as more complex models for mRAP prediction (p>0.05). CMR-derived mRAP ≥10 mm Hg was better associated with outcomes than mRAP ≥8 mm Hg in the clinical cohort (n=101) with diagnostic power for peripheral oedema (area under the curve (AUC) 0.75, p=0.02) and heart failure hospitalisation (AUC 0.93, p<0.01). Kaplan-Meier analysis demonstrated elevated CMR-derived mRAP (≥10 mm Hg) was associated with reduced survival compared with mRAP <10 mm Hg (χ^2^=5, p=0.02) over a mean follow-up of 6.8 years.

**Conclusion:**

mRAP can be estimated by CMR. Raised CMR-derived mRAP is predictive of lower-limb oedema, heart failure hospitalisation and all-cause mortality.

**WHAT IS ALREADY KNOWN ON THIS TOPIC**

- Right atrial pressure (RAP) is a measure of cardiac preload with elevated pressures being associated with adverse clinical outcomes.
- RAP can be measured invasively or with ultrasound, but despite the increasing use of cardiac MRI (CMR), no model currently exists to derive RAP from CMR.

**WHAT THIS STUDY ADDS**

- This study shows that RAP can be estimated from simple measurements of the right atrium on CMR with reasonable accuracy to determine whether RAP is elevated or not.
- It demonstrates in an independent population that elevated CMR-derived RAP is associated with poorer clinical outcomes.

**HOW THIS STUDY MIGHT AFFECT RESEARCH, PRACTICE OR POLICY**

- This study acts as a proof of principle.
- Further studies involving a broader range of patients, as well as direct validation using multiple methods of RAP measurement within the same cohort, are required.
- These will allow us to refine the model, improve its accuracy on an individual patient level and use CMR-derived RAP as a metric in calculating cardiac power output, gauging response to treatment and assessing its value in predicting prognosis.

## Introduction

 Right atrial pressure (RAP) is a crucial haemodynamic parameter in diagnosing and managing cardiovascular diseases. It serves as a surrogate for right ventricular filling pressure (preload) and guides fluid management.^1^ Traditionally, RAP has been approximated by central venous pressure (CVP), measured either invasively with a catheter in the superior vena cava or non-invasively using bedside ultrasound. This information aids in prognostic stratification of patients with heart failure and pulmonary hypertension (PH). CVP trends, combined with cardiac output measurements, offer valuable insights for optimising fluid management and haemodynamics. Therefore, accurate RAP estimation holds significant clinical value to guide therapeutic interventions.^2^

Non-invasive imaging techniques have emerged as safer and more accessible alternatives to invasive methods for estimating RAP.^3^ Among these, ultrasound assessment of the size and collapsibility of the inferior vena cava (IVC) is most commonly used for RAP estimation, but is susceptible to operator-dependent and patient-dependent variability.^4^

Recent studies suggest that cardiovascular magnetic resonance (CMR) imaging offers superior capabilities for estimating left ventricular filling pressure compared with echocardiography.^5^ This advantage stems from CMR’s inherent strengths, including reduced operator dependence, full three-dimensional visualisation for four-chamber volumetric assessments, tissue characterisation and quantification of valvular disease complementary to echocardiography. These findings raise the possibility that CMR-based volumetric assessment of the right heart, particularly the right atrium (RA), could accurately estimate mean RAP (mRAP), something which is not currently possible with CMR.

This study aimed to address this hypothesis by leveraging a large dataset of paired CMR and invasive right heart catheter (RHC) data acquired on the same day from patients being evaluated for dyspnoea. We first developed a model for estimating mRAP using CMR volumetric measurements and patient characteristics. Subsequently, this model was applied to an external cohort of patients without paired RHC data but with longitudinal data on clinical outcomes, to assess the value of CMR-derived mRAP in predicting clinical outcomes.

## Methods

This study is not a development and subsequent external validation of a predictive model of RAP due to the absence of RAP data available in the clinical cohort. As such, the Transparent Reporting of a multivariable prediction model for Individual Prognosis or Diagnosis (TRIPOD) guidelines do not fully apply. We have attempted to adhere to the TRIPOD guidelines where possible within the remit of the specific journal guidance.^6^ Complete case analysis from available data was used in this hypothesis-generating study.

### Study cohort

Two existing registry cohorts were used. For development, a well-defined cohort of 672 individuals from the ASPIRE (Assessing the Spectrum of Pulmonary Hypertension In a REferral Centre) registry (NHS IRAS ID: 211400) referred to Sheffield Teaching Hospitals for the evaluation of dyspnoea over an 8-year period from 2012 to 2020 was used. All patients underwent RHC and CMR, with procedures performed within 24 hours of each other. The inclusion criteria were signs and symptoms of heart failure, age >18 years, reasonable CMR quality (in particular, four-chamber cines without any foreshortening and short-axis cine images) and the provision of informed consent. The exclusion criteria included a subsequent diagnosis of pulmonary arterial hypertension (PAH) type 1, as well as contraindications to RHC or CMR, such as claustrophobia and end-stage heart failure.^5^ Specifically, patients with raised pulmonary pressures related to left heart disease were not excluded from this cohort.

The clinical outcomes cohort consisted of retrospective CMR data collected at Norfolk and Norwich University Teaching Hospitals. To be eligible for inclusion in the registry, patients had to be at least 18 years of age and have a scan of sufficient quality for segmentation purposes. We collected data for this work from the year 2016. Consecutive patients undergoing CMR were included.

Exclusion criteria were body weight exceeding 120 kg, inability to lie flat, pregnancy, presence of incompatible devices or implants and any other contraindications to CMR. These contraindications were, but are not limited to, allergy to the contrast agent, claustrophobia and end-stage renal impairment, defined as an estimated glomerular filtration rate of <30 mL/min.

### Ethical approval and consent to participate

For the Sheffield ASPIRE left heart disease cohort, ethical approval for the most recent research protocol was granted by the National Research Ethics Service (16/YH/0352, approval date 31 October 2016), but the registry has been running since 2001.^7^ In the case of the Norwich cohort, ethical approval was deemed unnecessary due to the retrospective nature of the data collection by the National Research Ethics Service. No specific study protocol was prepared for this retrospective analysis. This study was conducted according to the principles outlined in the Declaration of Helsinki (V.2013).

### Patient or public involvement

There was no patient or public involvement in the design of this study.

### Cardiac magnetic resonance protocol

At Sheffield, CMR was performed with a 1.5 T GE HDx scanner (GE Healthcare, Milwaukee, Wisconsin, USA). The protocol included two-chamber, three-chamber, four-chamber and short-axis cine acquisitions using a retrospective cardiac-gated multislice steady-state free precession sequence (tricuspid regurgitation (TR) 2.8 ms, TE (Echo Time) 1.0 ms, flip angle 50°, field of view 48×43.2, 256×256 matrix, 125 kHz bandwidth and slice thickness 8–10 mm). The short-axis cine images were used to measure left ventricular (LV) end-diastolic volume (LVEDV), LV end-systolic volume (LVESV), right ventricular (RV) end-diastolic volume (RVEDV) and RV end-systolic volume (RVESV). RA end-systolic volume (RAESV), RA end-diastolic volume (RAEDV) and RA strain were measured using the four-chamber cines.

From end-diastolic and end-systolic volumes, LV stroke volume (LVSV), LV ejection fraction (LVEF), RV stroke volume (RVSV), RV ejection fraction (RVEF), RA stroke volume (RASV) and RA ejection fraction (RAEF) were calculated.

At Norwich, CMR was performed on a 1.5 T Magnetom Sola (Siemens Healthineers, Erlangen, Germany). The CMR protocol was similar to the Sheffield derivation cohort, with particular attention to the long-axis and short-axis cines. As previous studies have shown good agreement for cines between different scanners, this was not considered an issue.^8^

#### CMR analysis

**Figure 1:**
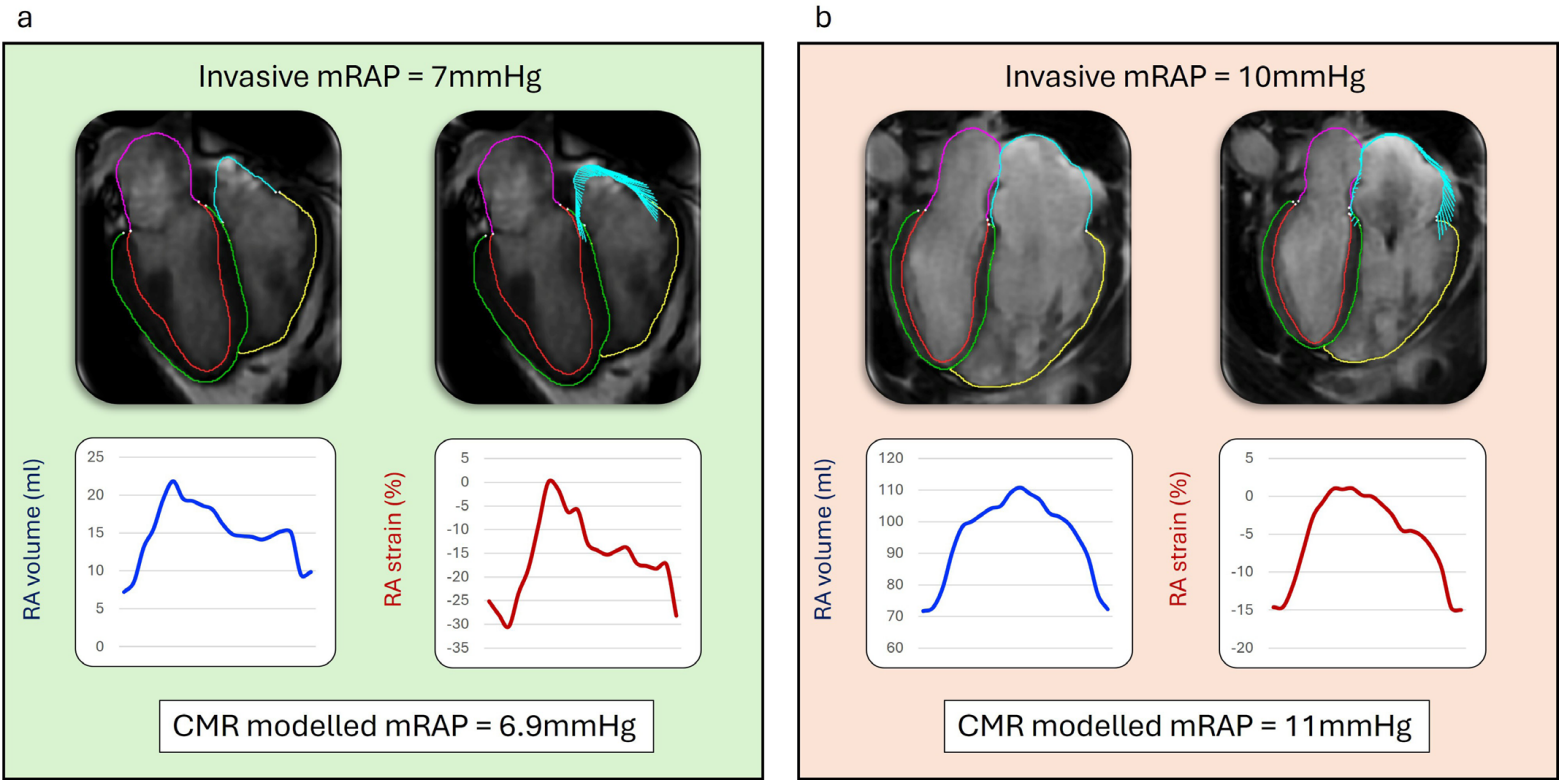
Two case examples from the study. (**a**) Case example of normal invasive right atrial pressure (RAP of 7 mm Hg). (**b**) Case example of a patient with a dilated right atrium and significantly compromised right atrial function (volume curves are flattened) with raised invasive mean RAP (mRAP) of 10 mm Hg. CMR, cardiovascular magnetic resonance.

All analyses used MASS research software (MASS, V.2023-EXP, Leiden University Medical Center, Leiden, The Netherlands). Both short-axis and long-axis time-resolved cine segmentation of the LV, RV and RA were done using artificial intelligence models previously described.^9 10^ For the RA strain, we recorded the minimum RA strain demonstrated (figure 1).

For quality control, the artificial intelligence-generated segmentations and time-resolved volume curves throughout all cardiac phases for all four chambers were evaluated by one of the authors (HA) and double-checked by an experienced clinician (SCMR (Society of Cardiovascular Magnetic Resonance) level 3).

### Clinical data

Baseline blood results were measured up to 3 months prior to CMR. In the Norwich registry arm of the study, the presence of peripheral oedema was assessed by the supervising clinician at the time of CMR with no blinding to clinical details. Heart failure admissions and mortality were assessed through longitudinal registry data collection using medical notes at Norfolk and Norwich NHS Foundation Trust. This was performed at a single time point (5 May 2023); therefore, individual patients had differing durations of follow-up. An episode of hospitalisation for heart failure that led to death was counted as both hospitalisation and death separately. No formal study follow-up was arranged.

### Statistical analysis

The primary outcome of interest was the accuracy of mRAP prediction by different CMR models within the derivation cohort. Secondary end points were the association of CMR mRAP, with all-cause mortality, hospitalisation and peripheral oedema at the time of CMR in the clinical cohort. Data were analysed using MedCalc Statistical Software, V.22.014 (MedCalc Software, Ostend, Belgium). Continuous variables are presented as the mean and SD. Discrete data are presented as numbers (n) and percentages (%). All data were treated as parametric. Variables were compared between groups using an independent samples t-test. The Youden Index was used to determine cut-off values. Kaplan-Meier analysis and Cox proportional hazards regression were used for multivariate analysis of prognosis. The total number of distinct outcomes was measured as the sum of clinical outcomes in heart failure—lower limb oedema, heart failure hospitalisation and death—on a per-patient basis during the study. Statistical significance was set at p<0.05. To evaluate the diagnostic performance of all four models within the derivation cohort, we conducted a receiver operating characteristic (ROC) analysis. Pairwise comparisons of ROC curves were performed using DeLong’s test to assess each model’s relative diagnostic accuracy and internally validate each model. Missing data were avoided for model development, and no interpolation techniques were used.

### Model development

We employed iterative methodologies to develop and validate the mRAP model, using invasively measured mRAP as the reference standard. To explore the correlation between invasive mRAP and CMR metrics, Pearson’s product-moment correlation coefficient (r) was computed. Potential predictor variables were selected based on empirical physiology with no previous models being available for reference. When variables exhibited high collinearity and were understood to have similar physiological meaning, the variable with the most physiological significance was chosen for further regression analysis. We deliberately restricted our findings to the six most correlated parameters, with right atrial dimensions demonstrating the strongest association with mRAP. Consequently, we constructed an mRAP model based on dimensional parameters using ‘stepwise’ multivariable regression (model 1). This dimensional model offers the advantage of being applicable on any Picture Archiving and Communication System Digital Imaging and Communications in Medicine system without the need for specialised software for postprocessing cardiac cine images.

Additionally, we developed a second model incorporating right atrial dimensions and right atrial deformation using multivariable ‘stepwise’ regression (model 2). A third model was created using right atrial and RV dimensions in multivariable ‘stepwise’ regression (model 3). Recognising that clinical characteristics, such as body surface area and sex, can influence chamber sizes and function, we also tested models that integrated these variables against the ground truth using the ‘enter’ method in multivariate regression (model 4).

## Results

### Derivation cohort

#### Demographics and CMR characteristics

The derivation cohort (n=672) was divided based on invasive mRAP: mRAP ≤8 mm Hg (44%) and mRAP >8 mm Hg (56%) (table 1). The threshold of 8 mm Hg was selected based on previous work indicating poorer prognosis in a variety of patient populations with an mRAP >8 mm Hg.^2 11^ The group with mRAP >8 mm Hg was significantly older (68±12 vs 64±14 years, p<0.01) and was more likely to be male (46% vs 37%, p=0.02). Other differences between the groups were observed in diastolic blood pressure (79±13 vs 76±11 mm Hg, p=0.01), LVESV (40±22 vs 34±17 mL, p=0.0004), LVEF (65±11% vs 69±10%, p=0.01), LV mass (101±34 vs 91±28 g, p<0.0001) and several other CMR metrics and invasive haemodynamic metrics. No significant differences were found in the proportion of patients with heart failure with preserved ejection fraction (HFpEF), heart failure with mildly reduced ejection fraction or heart failure with reduced ejection fraction respectively, or heart rate, systolic blood pressure, LVEDV and LVSV.

**Table 1:** Study demographics of the derivation cohort (n=672)

	mRAP ≤8 mm Hg	mRAP >8 mm Hg	P value
N (%)	295 (44%)	377 (56%)	
Age (years)	64±14	68±12	<0.01
Male sex (%)	109 (37%)	174 (46%)	0.02
HFpEF (%)	150 (51%)	195 (52%)	0.82
HFmrEF (%)	7 (2.4%)	18 (5%)	0.10
HFrEF (%)	5 (1.7%)	10 (3%)	0.40
Heart rate (bpm)	72±15	70±16	0.05
Systolic blood pressure (mm Hg)	142±25	143±27	0.52
Diastolic blood pressure (mm Hg)	76±11	79±13	0.01
CMR metrics			
LVEDV (mL)	108±33	113±40	0.10
LVESV (mL)	34±17	40±22	<0.01
LVSV (mL)	74±23	73±25	0.76
LVEF (%)	69±10	65±11	<0.01
LV mass (g)	91±28	101±34	<0.01
RVEDV (mL)	127±49	166±64	<0.01
RVESV (mL)	69±38	100±52	<0.01
RVSV (mL)	57±22	66±29	<0.01
RVEF (mL)	47±12	42±14	<0.01
RAEDV (mL)	69±36	122±68	<0.01
RAESV (mL)	37±30	87±63	<0.01
RA SV (mL)	32±14	35±17	0.01
RAEF (%)	50±15	35±17	<0.01
RA peak strain (%)	−25±9	−17±9	<0.01
CMR-derived mRAP (mm Hg)	9±2	12±4	<0.01
Invasive haemodynamic metrics			
Mean pulmonary artery pressure (mm Hg)	31±12	44±12	<0.01
Mean pulmonary capillary wedge pressure (mm Hg)	10±4	17±6	<0.01
mRAP (mm Hg)	5±2	14±5	<0.01

CMR, cardiovascular magnetic resonance; EDV, end-diastolic volume; EF, ejection fraction; ESV, end-systolic volume; HFmrEF, heart failure with mildly reduced ejection fraction; HFpEF, heart failure with preserved ejection fraction; HFrEF, heart failure with reduced ejection fraction; LV, left ventricular; mRAP, mean right atrial pressure; RA, right atrial; RV, right ventricular; SV, stroke volume.

#### CMR correlations with invasive mean right atrial pressure

**Figure 2:**
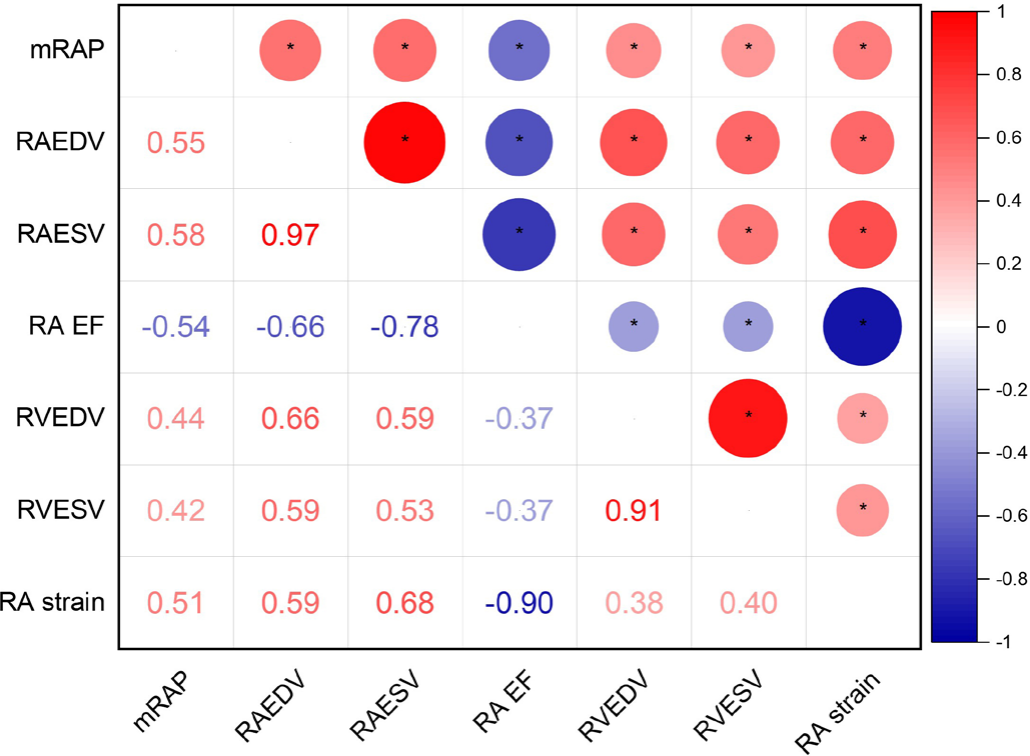
(**a**) Correlogram of invasive mRAP to several CMR right heart indices. (**b**) Scatter plot with heat map showing the association between RAESV and invasive mRAP. *P≤0.05. CMR, cardiovascular magnetic resonance; mRAP, mean right atrial pressure; RAEDV, right atrial end-diastolic volume; RAEF, right atrial ejection fraction; RAESV, right atrial end-systolic volume.

In the derivation cohort, several CMR indices showed significant correlations with invasive mRAP (mm Hg). The strongest correlations were observed with RAESV (mL) (0.58, p<0.01), followed by RAEDV (mL) (0.55, p<0.01) and RAEF (%) (−0.55, p<0.01) (online supplemental table 1). RA peak strain also showed a strong correlation (0.51, p<0.01). RVEDV (mL) (0.44, p<0.01) and RVESV (mL) (0.42, p<0.01) had moderate correlations. Other significant but less strong correlations were observed with RVSV (mL) (0.28, p<0.01), RVEF (mL) (−0.22, p<0.01), LV mass (g) (0.17, p<0.01) and LVESV (mL) (0.14, p=0.02). LVEF (%) also showed a significant correlation (−0.15, p=0.01) (figure 2). The correlations with RASV (mL) (0.09, p=0.03) and LVEDV (mL) (0.08, p=0.03) were statistically significant but weak. LVSV (mL) showed no significant correlation (0.01, p=0.86).

#### Multiple regression modelling of mRAP

Four models were generated for estimating mRAP (table 2). In model 1, the simple right atrial dimension model, RAESV was a significant predictor (coefficient=0.06, p<0.01). Model 2, which considered right atrial dimensions and strain, found both RAESV (coefficient=0.04, p<0.01) and peak right atrial strain (coefficient=0.13, p<0.01) to be significant predictors. In model 3, which incorporated right atrial dimensions and RV dimensions, RAEDV (coefficient=−0.03, p=0.02), RAESV (coefficient=0.08, p<0.01) and RVESV (coefficient=0.02, p<0.01) were all significant predictors. Notably, RAEDV was not included in models 1 and 2, and RVEDV was not included in model 3. In model 4, RAESV was adjusted for sex and body surface area. All three variables were statistically significant (p<0.05): sex with a coefficient of −1.21, RAESV with a coefficient of 0.06 and body surface area with a coefficient of 4.08.

**Table 2:** Four stepwise methods of multiple regression models for estimating mean right atrial pressure were generated

Independent variables	Coefficient	SE	t	P value
Model 1: simple right atrial dimension model				
(Constant)	6.4547			
RAESV	0.05828	0.003172	18.372	<0.01
Variables not included in the model: RAEDV				
Model 2: right atrial dimensions and strain model				
(Constant)	9.9835			
RAESV	0.04335	0.004269	10.154	<0.01
Peak RA strain	0.1274	0.02492	5.114	<0.01
Variables not included in the model: RAEDV				
Model 3: right atrial dimensions and right ventricular dimensions model				
(Constant)	6.138			
RAEDV	−0.02954	0.01216	−2.429	0.01
RAESV	0.07952	0.01267	6.277	<0.01
RVESV	0.02148	0.004568	4.703	<0.01
Variables not included in the model: RVEDV				
Model 4: patient corrected RAESV model				
(Constant)	−0.8179			
Sex	−1.2113	0.01216	−2.429	0.02
RAESV	0.05831	0.01267	6.277	<0.01
Body surface area	4.0842	0.004568	4.703	<0.01
Variables not included in the model				

EDV, end-diastolic volume; ESV, end-systolic volume; RA, right atrial; RV, right ventricular.

#### Performance of the four models: internal diagnostic checks

**Figure 3:**
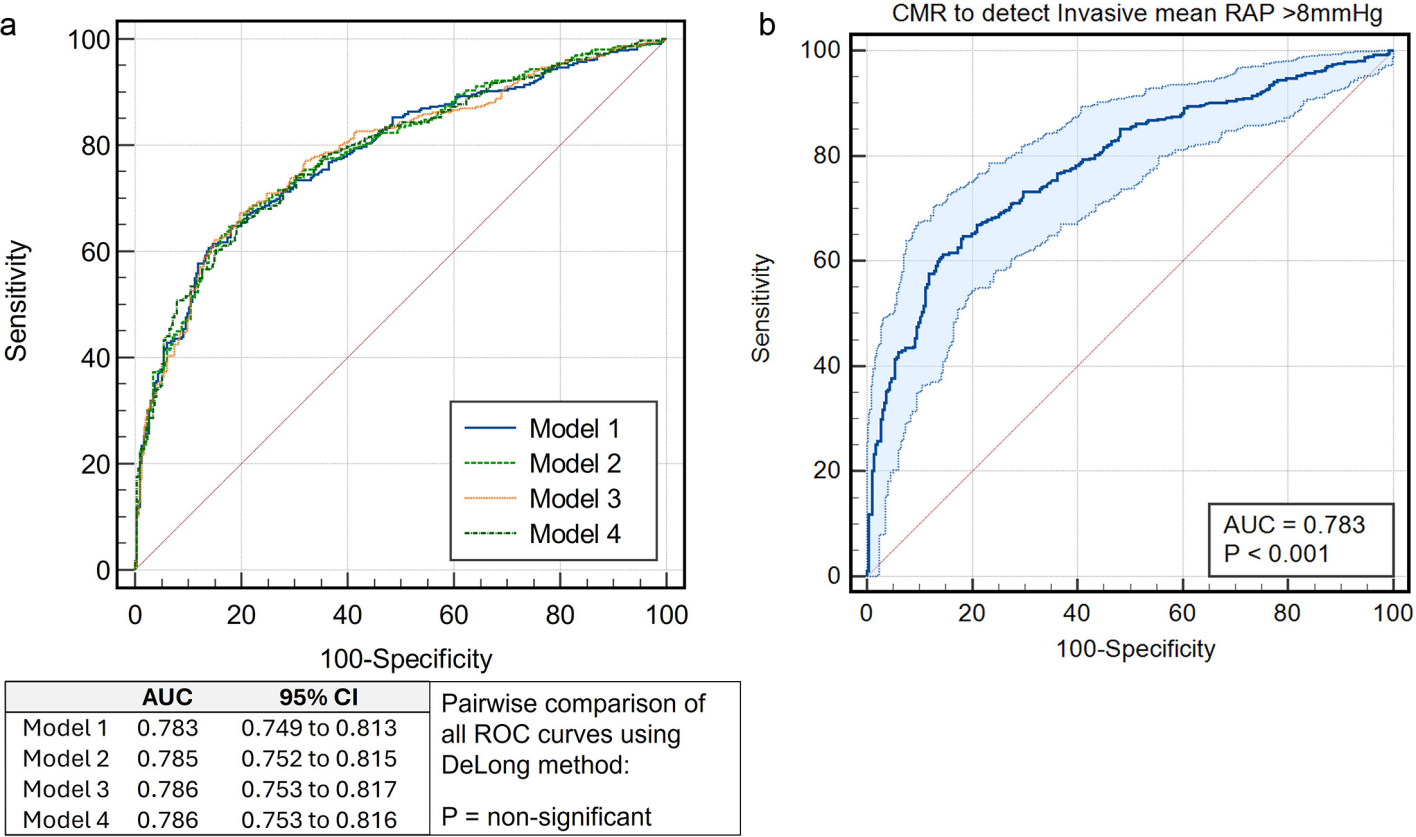
Internal validation checks. (**a**) Three physiological models to estimate invasive mRAP. Model 1 only incorporates RAESV, model 2 incorporates RAESV and peak strain, whereas model 3 incorporates RAESV and right ventricular volumes. Model 4 includes RAESV corrected for sex and body surface area. There is no significant difference in diagnostic power using models 2, 3 or 4 over model 1, which remains the simplest. (**b**) Model 1 ROC with 95% CI presented in light blue with an mRAP threshold of 8 mm Hg. AUC, area under the curve; mRAP, mean right atrial pressure; RAESV, right atrial end-systolic volume; ROC, receiver operating characteristic.

The performance of the models was assessed on the derivation cohort using ROC analysis before testing on the outcomes cohort (figure 3). Model 1 had an area under the curve (AUC) of 0.78, SE of 0.02 and a 95% CI from 0.75 to 0.81; model 2 with an AUC of 0.79, SE of 0.02 and a 95% CI from 0.75 to 0.82; model 3 with an AUC of 0.79, SE of 0.02 and a 95% CI from 0.75 to 0.82 and model 4 with an AUC of 0.79, SE of 0.02 and a 95% CI from 0.75 to 0.82. A pairwise comparison of ROC curves indicated no significant differences between the models (p>0.05 in all cases), suggesting similar predictive performance. As model 1 was the simplest with no difference in accuracy to more complex models, it was taken forward for use. The equation to estimate mRAP was: CMR mRAP (mm Hg)=6.4547+(RAESV×0.05828).

### Clinical outcomes cohort

#### Demographics

The demographics of the clinical outcomes cohort (n=101) are described in table 3. Sixteen patients (16%) died from any cause during the follow-up period. Patients who died were significantly older (67.8±9.7 vs 51.6±16.3 years, p=0.01), were more likely to be male (94% vs 60%, p=0.01) and had a higher incidence of ischaemic heart disease (56% vs 25%, p=0.01) and oedema (25% vs 7%, p=0.03). The baseline creatinine levels were also significantly higher in the deceased group (96.3±23.8 vs 81.9±23.3 µmol/L, p=0.03).

**Table 3:** Demographics of the clinical outcomes cohort (n=101)

Demographics	Alive (n=85)	Dead (n=16)	P value
Age, years	51.6±16.3	67.8±9.7	<0.01
Male sex, n (%)	51 (60)	15 (94)	0.01
Body surface area, m^2^	1.95±0.21	2.05±0.20	0.09
Smokers, n (%)	28 (33)	5 (31)	0.87
Hypertension, n (%)	24 (28)	7 (44)	0.22
Diabetes mellitus, n (%)	7 (8)	3 (19)	0.2
Atrial fibrillation, n (%)	13 (15)	5 (31)	0.13
Ischaemic heart disease, n (%)	21 (25)	9 (56)	0.01
Oedema, n (%)	6 (7)	4 (25)	0.03
Haemoglobin, g/L	126.7±43	146.7±18	0.07
Creatinine, μmol/L	81.9±23.3	96.3±23.8	0.03
Urea, mmol/L	7.2±9.4	7.1±2.5	0.99
CMR mRAP >10 mm Hg	10 (12%)	5 (31%)	0.04

CMR, cardiac magnetic resonance; mRAP, mean right atrial pressure.

There were, however, no significant differences in body surface area, smoking status, hypertension, diabetes mellitus, atrial fibrillation (AF), haemoglobin levels and urea levels between the two groups.

#### Association of CMR mRAP with clinical outcomes

**Figure 4:**
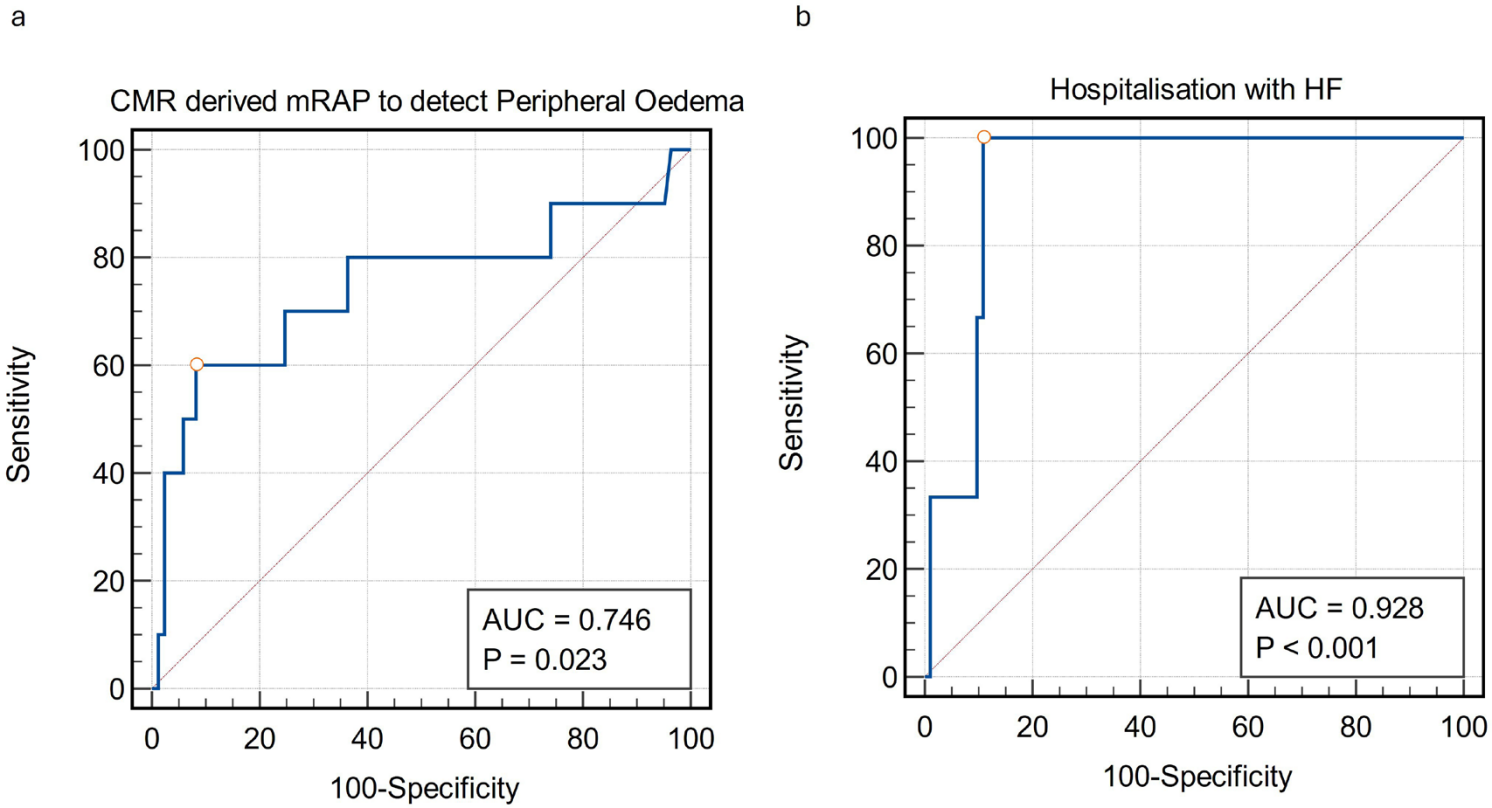
Clinical outcomes cohort results. (**a**) ROC curve of CMR-derived mRAP to detect peripheral pitting oedema in patients (events=10). (**b**) ROC curve of CMR-derived mRAP for hospitalisation (events=3). AUC, area under the curve; CMR, cardiovascular magnetic resonance; mRAP, mean right atrial pressure; ROC, receiver operating characteristic.

CMR-derived mRAP was determined using the model developed in the derivation cohort. A higher percentage of the deceased group had CMR mRAP >10 mm Hg (31% vs 12%, p=0.04) compared with CMR mRAP >8 mm Hg (21% vs 14%, p=0.34). Hence, for further prognostic analysis, we used the higher threshold of CMR mRAP. From the total Norwich cohort, 10 patients (10%) had lower limb pitting oedema recorded at the index CMR scan. It was hypothesised that if CMR mRAP was reflective of truly elevated RAP, then patients would be more likely to present with peripheral oedema. The area under the ROC curve for mRAP against the presence of lower limb pitting oedema was 0.75 (SE=0.11, 95% CI 0.65 to 0.83, p=0.02), indicating a strong association (figure 4a). The Youden Index was 0.52, with an associated criterion of ≥10 mm Hg. The sensitivity and specificity using an mRAP ≥10 mm Hg were 60% and 92%, respectively.

Three patients were hospitalised for decompensated heart failure during the follow-up period (figure 4b). The AUC for mRAP was 0.93 (SE=0.04, 95% CI 0.86 to 0.97, p<0.01), indicating a significant predictive capability. The Youden Index was 0.89, with an associated criterion of ≥10 mm Hg. The sensitivity and specificity of this model were 100% and 89%, respectively, in the context of a low event rate.

#### Survival analysis

**Figure 5:**
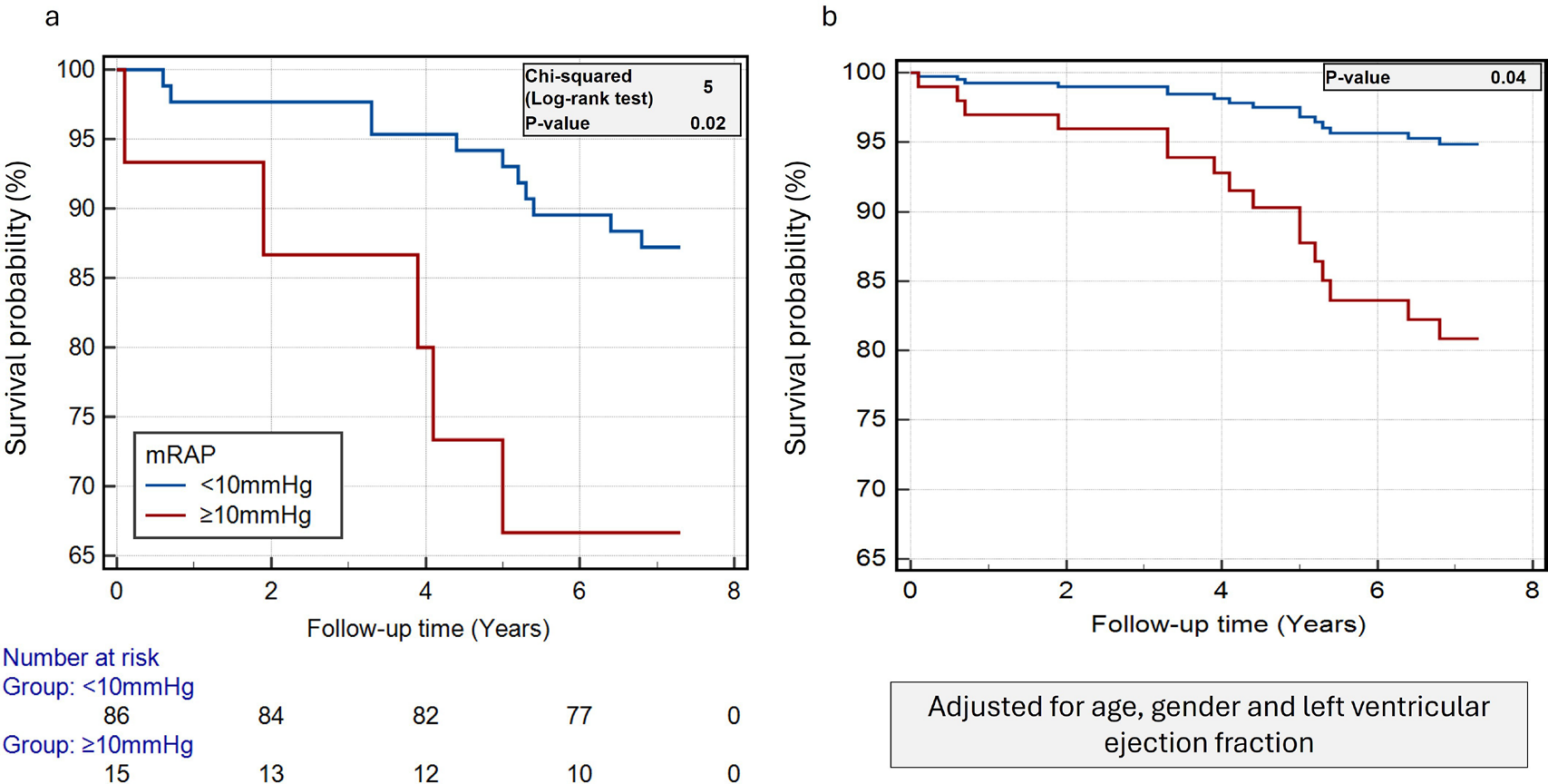
Clinical outcomes cohort to investigate the prognostic role of mRAP. (**a**) Kaplan-Meier curves demonstrate that CMR-derived mRAP >10 mm Hg is associated with an increased risk of all-cause mortality. (**b**) Cox-regression survival curves demonstrate that the risk of all-cause mortality remains high in patients with CMR-derived mRAP >10 mm Hg even after adjusting for age, sex and left ventricular ejection fraction. CMR, cardiovascular magnetic resonance; mRAP, mean right atrial pressure.

In the Kaplan-Meier analysis, the mean follow-up was 6.8 years. The mean survival time for patients with CMR mRAP <10 mm Hg was 7 years compared with 6 years for patients with CMR mRAP ≥10 mm Hg (log-rank χ^2^=5, p=0.02) (figure 5).

After adjusting for age, sex and LVEF in Cox-regression analysis, CMR mRAP ≥10 mm Hg still demonstrated poorer outcomes versus patients with normal mRAP (HR 4.02, p=0.04) (figure 5b).

#### Cumulative heart failure outcomes

**Figure 6:**
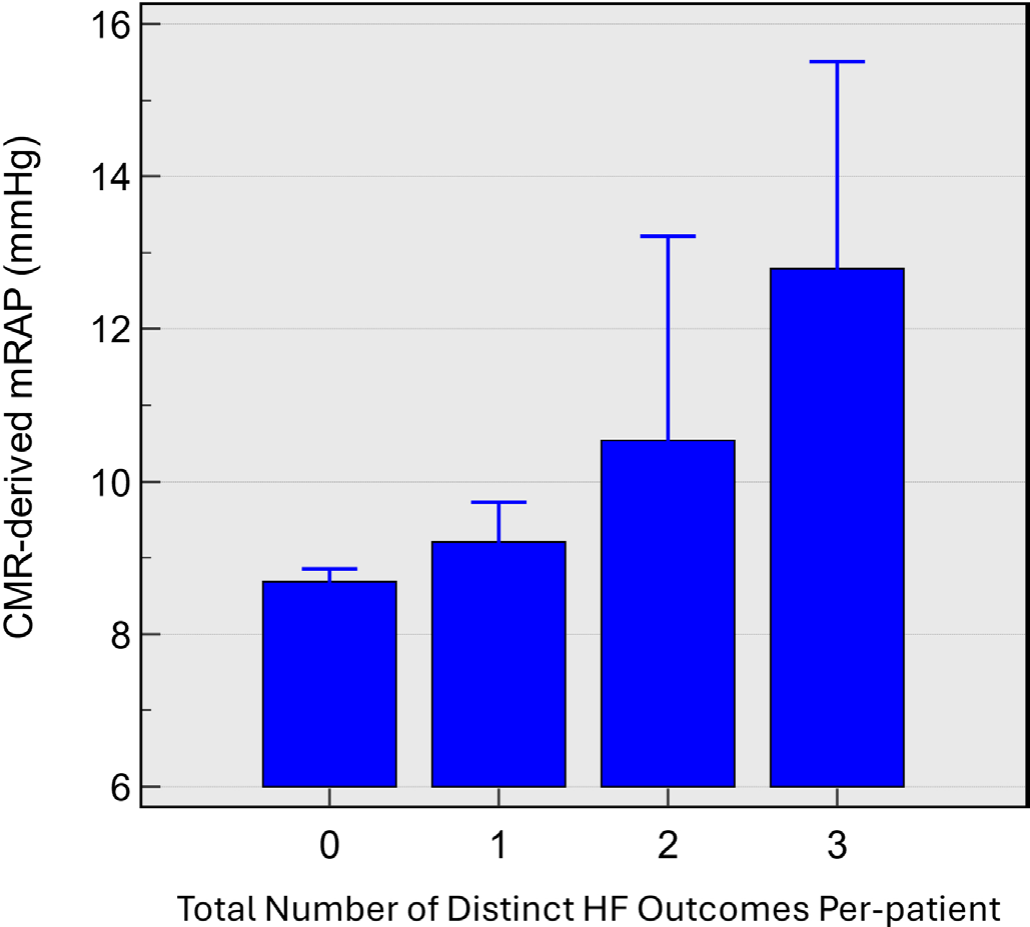
Bar chart of CMR-derived mRAP (y-axis), stratified by the number of outcomes patients experienced (oedema, hospitalisation, death); 0 being the absence of any outcome, 3 being suffering all measured adverse outcomes. CMR, cardiovascular magnetic resonance; HF, heart failure; mRAP, mean right atrial pressure.

The CMR mRAP increases with the accumulation of heart failure outcomes, specifically, 8.7±2 mm Hg for no outcomes, 9.2±2 mm Hg for one adverse outcome, 10.5±4 mm Hg for two adverse outcomes and 12.8±4 mm Hg for three adverse outcomes, indicating a trend of increasing mRAP as the number of heart failure outcomes accumulate (p=0.005) (figure 6).

## Discussion

The primary aim of this study was to develop a model to estimate mRAP using CMR. The main finding of this study is that CMR can estimate mRAP with moderate correlation with invasive RHC, and the key variables associated with RAP are RA end-systolic and end-diastolic volumes, RA peak strain and RV end-systolic volume. In a separate clinical cohort of patients receiving CMR for broad clinical indications, increased CMR-derived mRAP is associated with lower limb pitting oedema, hospitalisation and all-cause mortality in a stepwise fashion.

### mRAP pathophysiology and association with outcomes

In the context of PAH, elevated mRAP measured by cardiac catheterisation has been identified as an independent risk factor for mortality.^12^ Furthermore, an elevation of RAP by echocardiography at baseline assessment was strongly associated with an increased risk of death or transplant in patients with PAH. Complementary to this, Leng *et al* demonstrated that CMR-assessed RA strain predicted decompensation in haemodynamics in PAH.^13^ Alenezi *et al* also demonstrated that right atrial function, including peak contraction strain, is independently associated with outcomes in PH.^1^ These studies highlight the common pathophysiological process of initially increased volume and pressure loading on the RA, leading to RA dilatation and reduced deformation. Our study builds on this evidence and demonstrates how these functional and volumetric parameters can be leveraged to estimate the RAP. Importantly, like Austin *et al*, we demonstrate the association of RAP with signs of right heart failure, hospitalisation and all-cause mortality.^12^

This study was not powered to determine the prognostic utility of the model; rather, the clinical cohort served to see if expected clinical outcomes are seen in patients in whom RAP is predicted to be elevated by CMR. In this cohort, an mRAP of 10 mm Hg was statistically associated with poorer outcomes differing from the 8 mm Hg threshold set in the development cohort. This is potentially consistent with literature finding that thresholds of up to 12 mm Hg are important to determine outcomes.^14^ Further fully powered work with an externally validated model will be required to determine the clinically significant threshold of RAP, which may differ by associated pathology.

### mRAP in HFpEF

In HFpEF, RAP is of significant clinical relevance as it can reflect the cumulative burden of abnormalities in the left heart, pulmonary vasculature and right heart.^2^ Elevated RAP in patients with HFpEF has been associated with more severe abnormalities in LV diastolic function as well as right heart structure and function. Furthermore, higher estimated RAP has been independently associated with an increased risk of cardiac deaths or heart failure hospitalisation, suggesting its utility for risk stratification in patients with HFpEF.^2^ In our derivation cohort, almost half of the patients had HFpEF (n=345). This improves the clinical relevance of using this method in patients with HFpEF, especially in identifying PH secondary to HFpEF, which is associated with poorer outcomes. Also, in the context of PH secondary to HFpEF, increased RAP and RA stiffness have been observed, which are thought to be a reflection of HFpEF severity rather than a sign of overt RV failure.^15^ This suggests that RAP could serve as a marker of disease severity in patients with HFpEF.

Moreover, in patients with PH due to left heart disease and HFpEF, the prognostic performance of cardiac power output (CPO), a powerful predictor of adverse outcomes in heart failure, was found to be improved when corrected for RAP.^14^ The initial derivation of CPO included the difference between mean arterial pressure and RAP in the numerator before multiplying by cardiac output. However, due to inaccuracies in estimating RAP, it was dropped from the equation, although studies have demonstrated that RAP plays an important independent prognostic role in the CPO equation.^16^ The ability to estimate RAP from CMR could now improve our ability to calculate CPO.^17^

### Clinical implications of CMR-derived mRAP

To the best of our knowledge, there was no existing CMR model to estimate the mRAP prior to this work, limiting the ability of CMR to provide a comprehensive assessment of haemodynamics. Our newly developed CMR model, which estimates mRAP, relies directly on the volumetric assessment of the RA. This assessment can be routinely performed either by manually segmenting the RA area at the end-systolic phase or by employing a time-resolved segmentation process facilitated by an artificial intelligence solution. Such solutions are readily available from most common software vendors.

Used in conjunction with the previously published model for pulmonary capillary wedge pressure (PCWP), the measurement of mRAP could enhance diagnostic accuracy. This is particularly beneficial in cases of PH associated with HFpEF and in patients with primary PAH, where PCWP is normal.^518^,^21^

Furthermore, haemodynamic variables such as systolic and diastolic systemic pressures can also be estimated by CMR. This allows for a more comprehensive, non-invasive haemodynamic assessment using CMR.^22^ Thus, our CMR model fills a critical gap in the field and paves the way for more advanced and precise non-invasive cardiovascular assessments.

### Limitations

This study does have limitations. First, there is potential for selection bias in the derivation cohort, which comprised patients referred to a tertiary centre for assessment of possible PAH due to symptoms of shortness of breath where no alternative cause had been identified by their referring institute. The ASPIRE registry therefore contains a greatly increased frequency of patients subsequently diagnosed with PAH compared with the general population undergoing CMR. It does, however, include a large cohort of patients in whom PAH was not identified. In provisional analysis, right-sided chamber dimensions and pressures were grossly different in patients subsequently diagnosed with PAH. To produce an initial model more reflective of the general cohort attending for CMR, we excluded patients subsequently diagnosed with PAH, therefore avoiding this selection bias in our cohort, but limiting the generalisability of findings. We intend to assess the prediction of mRAP by CMR on patients with PAH in the future.

The clinical cohort was more inclusive, disease-agnostic and smaller, with relatively few clinical outcomes. Peripheral oedema is a subjective and non-specific presentation; however, it is frequently associated with heart failure and elevated right heart pressures. Consequently, the prognostic assessment derived from this study necessitates further validation through larger, disease-specific studies to enhance its applicability and accuracy. Another limitation of our study was the absence of echocardiography data. We did not incorporate echocardiography into our routine study protocol, and as a result, these data including echo-derived right-sided pressures and measurements and valvular function were unavailable for comparative analysis. The CMR protocol used for the ASPIRE registry also lacked the pulmonary arterial flows required to compute TR, and therefore this potentially important predictor variable is unavailable in our model. The same is true for the presence of AF. While the frequency of these is not available, patients recruited into ASPIRE were referred for evaluation of breathlessness where no alternative cause was readily identified; therefore, both significant TR and AF are likely to be relatively infrequent.

The same is true for RHC assessment in the outcomes cohort. Both cohorts also reflected a population unlikely to be hypovolaemic. This may contribute to the model’s accuracy and discriminatory power being increased at higher RAP with lower accuracy at normal or low RAP. Further studies, including healthy volunteers or those with reduced circulating fluid volumes, would be required to develop a more accurate model at lower RAPs.

In summary, while our study provides valuable insights, further research is warranted. Specifically, future studies should investigate the role of mRAP in disease-specific outcomes to enhance our understanding and potentially improve patient management strategies.

## Conclusion

mRAP can be estimated by CMR four-chamber right atrial end-systolic volume. Elevated CMR-derived mRAP is associated with signs of right heart failure, hospitalisation due to heart failure and all-cause mortality.

## Supplementary material

10.1136/openhrt-2025-003216 online supplemental file 1

## Data Availability

Data are available on reasonable request.
