# Supplementary material for: Cardiac MRI-derived mean right atrial pressure and its prognostic importance
Source: Open Heart. 2025 Jun 22;12(1):e003216. doi: 10.1136/openhrt-2025-003216 (PMC12184376; doi:10.1136/openhrt-2025-003216)
Supplement: online supplemental file 1 [file openhrt-12-1-s001.docx]

**Supplementary Table 1**. Pearson correlation of several CMR indices with invasive mean right atrial pressure (mmHg) in the derivation cohort.

|  | **Correlation coefficient** | **P-value** |
| --- | --- | --- |
| RAEDV (ml) | 0.553 | <0.01 |
| RAESV (ml) | 0.579 | <0.01 |
| RAEF (%) | -0.545 | <0.01 |
| RA SV (ml) | 0.085 | 0.03 |
| RA peak strain (%) | 0.511 | <0.01 |
| RVEDV (ml) | 0.442 | <0.01 |
| RVESV (ml) | 0.416 | <0.01 |
| RVSV (ml) | 0.257 | <0.01 |
| RVEF (%) | -0.223 | <0.01 |
| LVEDV (ml) | 0.084 | 0.03 |
| LVESV (ml) | 0.144 | <0.01 |
| LV SV (ml) | 0.007 | 0.85 |
| LVEF (%) | -0.148 | <0.01 |
| LV mass (gram) | 0.173 | <0.01 |
